# Conventional and Tropism-Modified High-Capacity Adenoviral Vectors Exhibit Similar Transduction Profiles in Human iPSC-Derived Retinal Organoids

**DOI:** 10.3390/ijms26010055

**Published:** 2024-12-24

**Authors:** Andrew McDonald, Carmen Gallego, Charlotte Andriessen, Michaela Orlová, Manuel A. F. V. Gonçalves, Jan Wijnholds

**Affiliations:** 1 Department of Ophthalmology, Leiden University Medical Center (LUMC), Albinusdreef 2, 2333 ZA Leiden, The Netherlands; a.mcdonald@lumc.nl (A.M.); c.a.andriessen@lumc.nl (C.A.);; 2 Department of Cell and Chemical Biology, Leiden University Medical Center (LUMC), Einthovenweg 20, 2333 ZC Leiden, The Netherlands; m.f.v.goncalves@lumc.nl; 3 Netherlands Institute for Neuroscience, Institute of the Royal Netherlands Academy of Arts and Sciences (KNAW), Meibergdreef 47, 1105 BA Amsterdam, The Netherlands

**Keywords:** adenoviral vector, high-capacity adenoviral vector, retinal organoids, gene therapy, photoreceptors, Müller glial cells

## Abstract

Viral vector delivery of gene therapy represents a promising approach for the treatment of numerous retinal diseases. Adeno-associated viral vectors (AAV) constitute the primary gene delivery platform; however, their limited cargo capacity restricts the delivery of several clinically relevant retinal genes. In this study, we explore the feasibility of employing high-capacity adenoviral vectors (HC-AdVs) as alternative delivery vehicles, which, with a capacity of up to 36 kb, can potentially accommodate all known retinal gene coding sequences. We utilized HC-AdVs based on the classical adenoviral type 5 (AdV5) and on a fiber-modified AdV5.F50 version, both engineered to deliver a 29.6 kb vector genome encoding a fluorescent reporter construct. The tropism of these HC-AdVs was evaluated in an induced pluripotent stem cell (iPSC)-derived human retinal organoid model. Both vector types demonstrated robust transduction efficiency, with sustained transgene expression observed for up to 110 days post-transduction. Moreover, we found efficient transduction of photoreceptors and Müller glial cells, without evidence of reactive gliosis or loss of photoreceptor cell nuclei. However, an increase in the thickness of the photoreceptor outer nuclear layer was observed at 110 days post-transduction, suggesting potential unfavorable effects on Müller glial or photoreceptor cells associated with HC-AdV transduction and/or long-term reporter overexpression. These findings suggest that while HC-AdVs show promise for large retinal gene delivery, further investigations are required to assess their long-term safety and efficacy.

## 1. Introduction

Viral vector delivery of gene therapy reagents holds great promise as an effective approach for treating a number of genetic diseases and has now been approved for several clinical therapies [1]. Primarily, the delivery vehicles have been adeno-associated viral vectors (AAV) owing to their desirable properties including low chromosomal integration and moderate stimulation of immune responses in the eye. In the context of retinal disease, AAV has been successfully utilized to deliver a *RPE65* transgene in the first clinically available retinal gene therapy, and a number of studies have shown AAV to be an effective vehicle for supplementation of retinal genes in several disease models [2,3]. Despite the popularity of AAV as a delivery vector, a substantial limitation consists of the relatively small packaging capacity of ~4.7 kb. This precludes the delivery of a number of large, yet clinically relevant retinal genes, such as *USH2A* (15.6 kb), *CDH23* (10.1 kb), *EYS* (9.4 kb), *CEP290* (7.4 kb), *GPR179* (7.1 kb), *ABCA4* (6.8 kb), *RP1* (6.5 kb), *MYO7A* (6.6 kb), or *CRB1* (4.2 kb) or gene editing components such as full-length prime editing (6.2 kb) and base editing (4.2–5.2 kb) constructs [4,5].

Alternative viral vectors for gene delivery consist of high-capacity human adenoviral vector (HC-AdV) particles, which are capable of carrying cargo up to 36 kb [6]. Indeed, the HC-AdV platform is able to deliver expression units encoding the human full-length dystrophin coding sequence (11.1 kb) to muscle cells [7]. These 3rd generation adenoviral vectors are devoid of all viral genes and have been shown to have a reduced cytotoxicity profile in vitro [8] and inflammatory response in vivo when compared to earlier generation adenoviral vectors deleted exclusively in a subset of viral regulatory functions [6,9]. The capsid shell of an AdV comprises multiple copies of three major capsid proteins (i.e., hexon, penton base, and fiber) and four minor cement proteins (i.e., IX, VIII, VI, and IIIa). In addition, six other proteins (i.e., μ, IVa2, V, VII, terminal protein, and adenovirus protease) are encapsidated along with the 36 kb dsDNA genome [10]. As there is an unmet need for vectors capable of delivering large retinal genes, we sought to determine the transduction efficiency and tropism of HC-AdV particles with unmodified and modified capsids in human retinal organoids.

Historically, the testing of gene therapy vectors has been performed in animal models, such as rodents and non-human primates [11,12]. Whilst these models offer a valuable platform for testing viral vectors in vivo, there are substantial differences between the tissues and organs of these animal models and those of humans, including the retina. In recent years, the development of human iPSC-derived retinal organoids has allowed for the production of organoid models capable of closely mimicking the human retina with a high degree of accuracy that, for instance, yield laminated retinal tissue containing all major retinal cell types [13,14].

Commonly used adenoviral vectors are based on the prototypic human adenovirus type 5 from species C, which engages the coxsackievirus and adenovirus receptor (CAR) to enter cells. In contrast, species B AdVs interact with different primary receptors that, depending on the type, mostly involves desmoglein 2 or CD46. In this study, we utilized an iPSC-derived retinal organoid model to assess the transduction efficiency and tropism of two HC-AdV vectors based on the classical AdV type 5 (HC-AdV5.F5) and a capsid-modified variant displaying fiber 50 domains (HC-AdV5.F50) capable of transducing CAR-negative cells by engaging with the broadly expressed CD46 receptor [15]. These two viral vectors contained sequences expressing a mCherry reporter gene under the control of the ubiquitous CAG promoter, and were applied to human retinal organoids at differentiation day (DD) 130. Strong transduction was observed throughout the retinal organoids, with efficient transduction of the photoreceptor layer and Müller glial cells being readily detected. Significantly, HC-AdV5.F5 and HC-AdV5.F50 vectors resulted in comparable transduction efficiencies and cell-type tropism profiles. Expression of the transgene was visible shortly following transduction and persisted until the termination of this study, namely, 110 days post-transduction. No detrimental effects were observed following HC-AdV transductions with organoid development and morphology aligning with non-transduced organoid controls.

Our results show the possibility of leveraging the HC-AdV system to deliver large genes in an efficient manner into both photoreceptors and Müller glial cells, and demonstrate the feasibility of using such vectors for the supplementation of large retinal genes which currently lack a suitable vector. A matter warranting further investigation, however, is the detection of swelling of the photoreceptor outer nuclear layer (ONL) at late timepoints after HC-AdV transduction of human retinal organoids.

## 2. Results

### 2.1. Adenoviral Vector Transduction of Retinal Organoids

**Figure 1:**
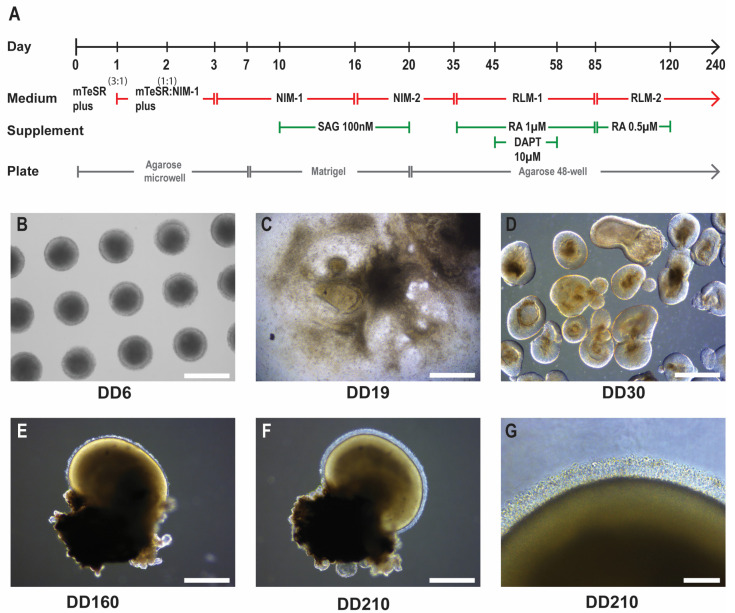
iPSC-derived retinal organoid differentiation. (**A**) Schematic of the retinal organoid differentiation timeline and culture conditions. Neural-induction medium 1 (NIM-1), neural-induction medium 2 (NIM-2), retinal lamination medium 1 (RLM-1), retinal lamination medium 2 (RLM-2), smoothened agonist (SAG), retinoic acid (RA), and gamma secretase inhibitor IX (DAPT). (**B**) Embryoid bodies formed from iPSCs in agarose microwells at DD6. Scale bar 500 µm. (**C**) Neuroepithelium formation on Matrigel at DD19. Scale bar 1 mm. (**D**) Isolated promising early-stage retinal organoid structures in floating culture at DD30. Scale bar 500 µm. (**E**) DD160 retinal organoid. Scale bar 500 µm. (**F**) Mature retinal organoid at DD210. Scale bar 500 µm. (**G**) Higher magnification image of inner/outer segment-like structures at DD210. Scale bar 100 µm.

To determine the feasibility of using HC-AdV vectors as gene delivery vehicles for retinal gene therapy, we utilized human iPSC-derived retinal organoids as a model to evaluate transduction efficiencies and study cell-type tropisms. Retinal organoids were differentiated from healthy donor iPSCs, using a modified differentiation protocol based on Zhong et al. [13] and Wahlin et al. [14] (Figure 1).

**Figure 2:**
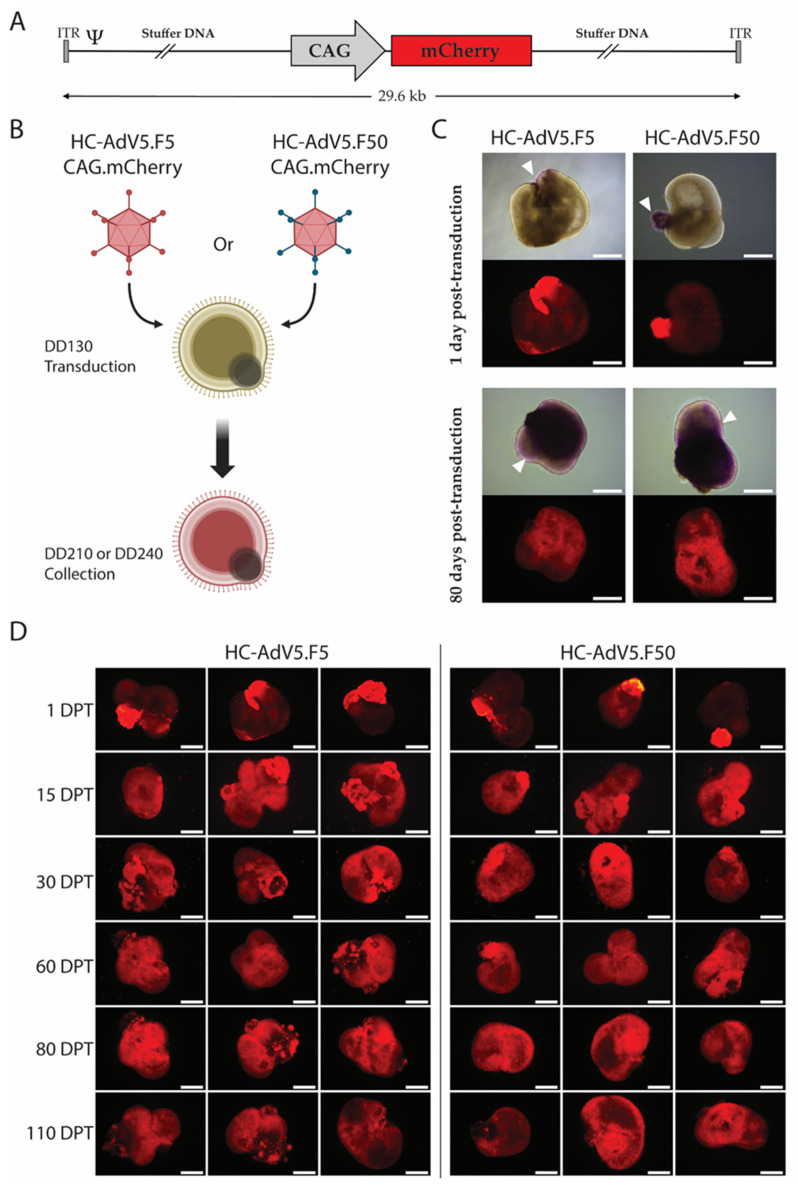
Adenoviral vector transduction of iPSC-derived retinal organoids. (**A**) A schematic representation of the HC-AdV vector genomes. Both HC-AdV5.F5 and HC-AdV5.F50 contain “stuffer” DNA and the reporter mCherry under the control of the hybrid CAG promoter. ITR and Ψ, adenoviral cis-acting inverted terminal repeat (origins of replication) and packaging signal elements necessary for, respectively, vector DNA replication and encapsidation in producer cells. (**B**) Schematic detailing the adenoviral vector transduction procedure in retinal organoids. Transduction occurs at DD130 with either HC-AdV5.F5 or HC-AdV50.F50 with collection and further analysis of the retinal organoids occurring at DD210 and DD240. Created with Biorender.com. (**C**) Representative live-cell fluorescence microscopy images of retinal organoids at 1 day and 80 days post-transduction. The mCherry reporter is visible in purple areas in the brightfield images (white arrows). Scale bars 500 µm. (**D**) Live-cell fluorescence microscopy analysis for mCherry expression in human retinal organoids. The retinal organoids were transduced with the indicated adenoviral vectors at DD130. Three representative images per time point are depicted. DPT = days post-transduction. Scale bars 500 µm.

We tested HC-AdV particles containing recombinant DNA packaged in prototypic AdV type 5 capsids or in modified AdV type 5 capsids displaying apical fiber motifs from AdV type 50 that, instead of CAR, engage CD46 on the cell surface [15,16]. These vectors will be hereafter referred to as HC-AdV5.F5 and HC-AdV5.F50, respectively. Both adenoviral vectors contained the same 29.6 kb DNA sequence encoding an mCherry reporter driven by a ubiquitous CAG promoter (Figure 2A). First, we sought to establish the optimal transduction time point and adenoviral vector dose. We tested three doses, i.e., 3.7 × 10^5^, 3.7 × 10^6^, and 3.7 × 10^7^ HeLa-transducing units (HTU). The transduction at 3.7 × 10^5^ and 3.7 × 10^6^ HTU did not result in efficient expression in the retinal organoids, whereas 3.7 × 10^7^ HTU was optimal at multiple time points, resulting in high level expression of the mCherry reporter within the retinal organoids. Initial testing conducted at differentiation day (DD) 130, 170, and 263, revealed that DD130 was the most suitable time point for adenoviral vector transduction of retinal organoids. Hence, following HC-AdV transduction at DD130, organoids were monitored up until DD210 or DD240 (Figure 2B) and, subsequently, the retinal organoids were cryopreserved and further analyzed by immunofluorescence confocal microscopy.

Live-cell imaging of transduced retinal organoids revealed transgene expression from day 1 post-transduction onwards with both vector types, demonstrating a rapid HC-AdV-driven transgene expression kinetics in these organoids (Figure 2C,D). mCherry expression was most pronounced in the dividing retinal pigment epithelium (RPE), a cell type well known for its phagocytic activity and rapid uptake of viral vector particles [17,18]. Remarkably, in the light microscopy images, purple coloration was visible in the RPE suggesting relatively high expression of the mCherry reporter (Figure 2C). Equally of note, the reporter was stably expressed up until the last collection timepoint of DD240. At DD210 and DD240, the mCherry signal intensity became more equal throughout the organoids than at earlier timepoints, with fluorescence signals being clearly visible in all areas of the organoids (Figure 2C,D). Importantly, transduction by HC-AdV particles appeared to have no effect on the regular differentiation of retinal organoids, as these organoids developed in a typical fashion, i.e., displayed a laminated structure with visible inner/outer segments in a “brush border” configuration (Figure 2C).

### 2.2. Transduction Efficiency of Adenoviral Vectors in Human Retinal Organoids

**Figure 3:**
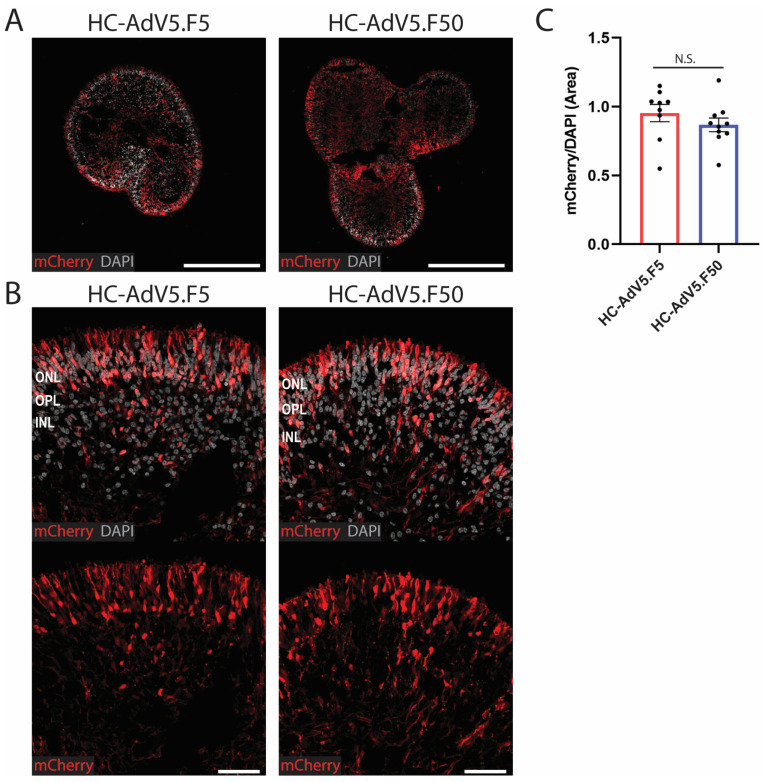
Transduction efficiency of HC-AdV5.F5 and HC-AdV5.F50 in human retinal organoids. (**A**) Representative images (10× magnification) of retinal organoids (DD210) following transduction at DD130 with either HC-AdV5.F5 or HC-AdV5.F50. Scale bars 500 µm. (**B**) Representative images (40× magnification) of retinal organoids (DD210) upon transduction at DD130 with either HC-AdV5.F5 or HC-AdV5.F50. Outer nuclear layer (ONL), outer plexiform layer (OPL), and inner nuclear layer (INL). Scale bars 50 µm. (**C**) Quantification of transduction efficiency in human retinal organoids using HC-AdV5.F5 and HC-AdV5.F50 calculated by mCherry-positive area normalized to DAPI-positive area. Each datapoint of the graph represents a single organoid, the value for each organoid is generated from the average value of three independent images at 40× magnification. Unpaired *t*-test; ns: not significant. Error bars represent the standard error of the mean (SEM). Number of individual organoids per condition: HC-AdV5.F5 *n* = 9, HC-AdV5.F50 *n* = 10.

After having established HC-AdV dosing and transduction timing, we sought to compare the transduction efficiencies achieved by using HC-AdV5.F5 versus HC-AdV5.F50. To this end, retinal organoids were transduced with 3.7 × 10^7^ HTU at DD130 and, subsequently, organoids were collected for analysis at either DD210 or DD240. Consistent with the previous data, transduction with HC-AdV vectors appeared to be efficient with strong mCherry signal detection observed throughout the organoid in both the inner and outer retina layers, although with regions of variable intensity (Figure 3A). In direct fluorescence microscopy images taken at 40× magnification it can be seen that both HC-AdV types transduced organoid regions in a largely similar manner, with many cells in the photoreceptor and inner retina layers expressing mCherry (Figure 3B).

Moreover, mCherry-positive Müller glial cell end feet projections could also be observed (Figure 3B). The quantification of transduction efficiencies was calculated by measuring the mCherry-positive area normalized to the DAPI-positive area to account for the size and density variations in individual retinal organoids. Statistically significant differences were not recorded, with HC-AdV5.F5 and HC-AdV5.F50 largely achieving comparable total transduction efficiencies at both DD210 (Figure 3C) and DD240 (Figure S1).

### 2.3. Adenoviral Vector Transduction of Müller Glial Cells

**Figure 4:**
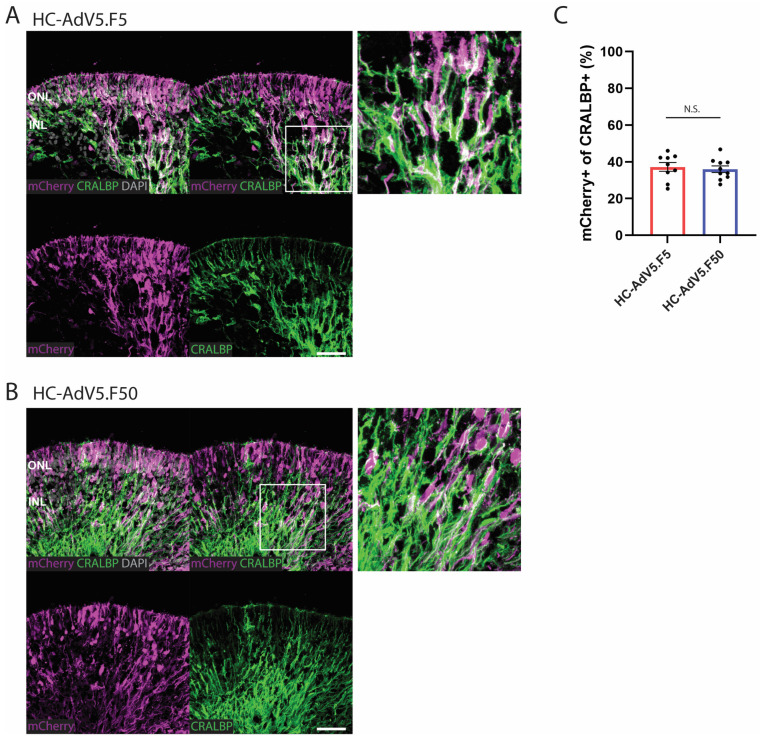
Adenoviral vector transduction of Müller glial cells. (**A**,**B**) Representative images of retinal organoids (DD210) following transduction with HC-AdV5.F5 and HC-AdV5.F50, respectively. Colocalization between the mCherry reporter and the Müller glial cell marker CRALBP is identified by white colored regions. Outer nuclear layer (ONL) and inner nuclear layer (INL). Scale bars 50 µm. (**C**) Quantification of transduced Müller glial cells, measured by the percentage of CRALBP-positive areas also expressing mCherry. Each datapoint of the graph represents a single organoid, the value for each organoid is generated from the average value of three independent images at 40× magnification. Unpaired *t*-test; ns: not significant. Error bars represent the standard error of the mean (SEM). Number of individual organoids per condition: HC-AdV5.F5 *n* = 9, HC-AdV5.F50 *n* = 10.

Following the analysis of total transduction levels achieved by regular and capsid-modified HC-AdV vectors, we next turned our attention to adenoviral vector transduction analysis in different retinal cell subtypes. First, we investigated the ability of HC-AdV5.F5 and HC-AdV5.F50 to transduce Müller glial cells. To this end, immunofluorescence microscopy analysis was used to detect the Müller glial cell marker CRALBP (Figure 4A,B). CRALBP staining can be seen in the inner retina with projections extending outwards to the ONL. Colocalization of CRALBP with mCherry was observed in many regions following transduction with either HC-AdV5.F5 or HC-AdV5.F50, as shown by the white overlap signal (Figure 4A,B). To quantify Müller glial cell transduction levels, we defined the Müller glial cells as the CRALBP-positive area and then determined which percentage of this area was also mCherry-positive. We found that HC-AdV5.F5 and HC-AdV5.F50 performed equally well in transducing the Müller glial cell area, with no statistically significant differences between the two adenoviral vector types being detected (Figure 4C). This result was consistently observed when analyzing vector-transduced specimens at DD240 (Figure S2).

### 2.4. Adenoviral Vector Transduction of Photoreceptors

**Figure 5:**
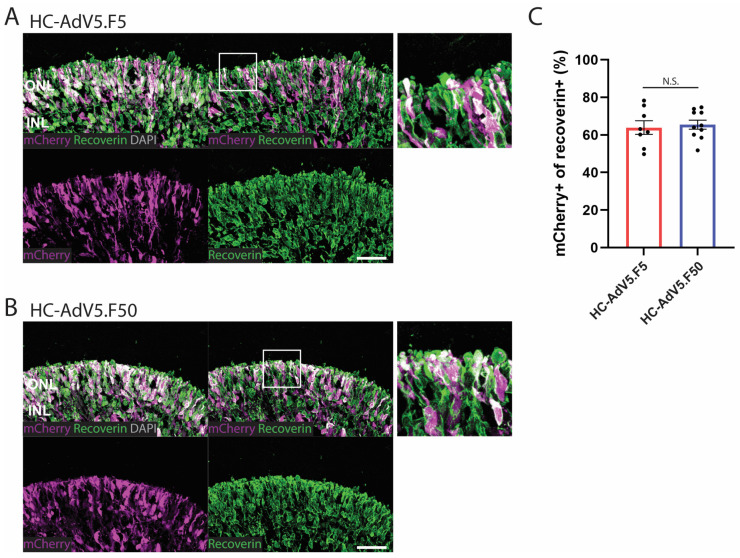
Adenoviral vector transduction of photoreceptors. (**A**,**B**) Representative images of retinal organoids (DD210) following transduction at DD130 with HC-AdV5.F5 and HC-AdV5.F50, respectively. Colocalization between the mCherry reporter and the photoreceptor marker recoverin is identified by white colored regions. Outer nuclear layer (ONL) and inner nuclear layer (INL). Scale bars 50 µm. (**C**) Quantification of transduced photoreceptors, measured by the percentage of the recoverin-positive area also expressing mCherry. Each datapoint of the graph represents a single organoid, the value for each organoid is generated from the average value of three independent images at 40× magnification. Unpaired *t*-test; ns: not significant. Error bars represent the standard error of the mean (SEM). Number of individual organoids per condition: HC-AdV5.F5 *n* = 8, HC-AdV5.F50 *n* = 10.

Next, we wished to determine the effectiveness of the two adenoviral vector types in transducing photoreceptors. To define the photoreceptors we made used of the pan-photoreceptor marker recoverin. In the same manner as to calculate the Müller glial cell transduction efficiencies, we defined the photoreceptor area as that positive for the recoverin marker. Although, in this case, we focused only on the ONL region as it is unclear whether other recoverin-positive cells in the inner retina are not the result of misplaced or immature photoreceptors or, possibly, ON bipolar cells. HC-AdV5.F5 and HC-AdV5.F50 transductions yielded a clear mCherry fluorescence signal in the ONL (Figure 5A,B), with colocalization of mCherry and recoverin seen in both the photoreceptor cell body and the protruding inner/outer segments (white areas). Quantification of mCherry located in the aforementioned photoreceptor area again revealed no significant differences between either adenoviral vector at DD210 (Figure 5C) or DD240 (Figure S3). The tropism of HC-AdV5.F50 in retinal organoids was also investigated using an alternative construct expressing EGFP driven by the human phosphoglycerate kinase 1 gene (PGK) promoter, with EGFP protein colocalization with CRALBP and recoverin being observed (Figure S4).

### 2.5. Increased ONL Thickness Following Adenoviral Vector Transduction

Although both adenoviral vector types yielded efficient and widespread transduction in the human retinal organoids, it was unclear whether the use of high vector doses, or the delivery of large quantities of constitutively active reporter expression units, result in any changes in retinal structure or, as an indicator of retinal stress, gliosis. The transduced and non-transduced organoids developed well-laminated retinal structures with visible inner/outer segment development emerging at the expected timepoints (Figure 2C). Additional immunofluorescence microscopy analysis showed a well-defined ONL and INL as seen by DAPI staining at 40× magnification (Figure 3). Although there was no visibly apparent impact of adenoviral vector transduction on the differentiation and structural organization of the organoids, we decided to investigate these aspects further.

**Figure 6:**
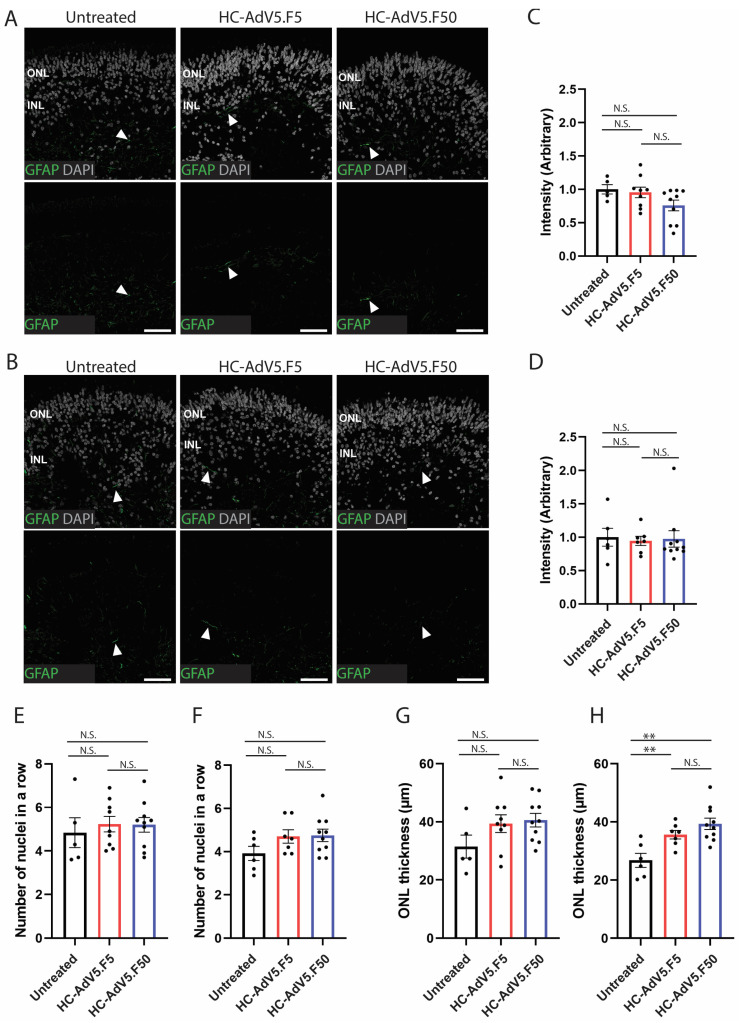
Increased thickness of the ONL observed at DD240 following HC-AdV transduction. (**A**) Representative images of GFAP expression in organoids at DD210 and (**B**) DD240 following adenoviral vector transduction (DD130). White arrows show GFAP-positive regions. Scale bars 50 µm. (**C**). Quantification of GFAP expression in DD210 and (**D**) DD240 organoids calculated by GFAP intensity normalized to DAPI area. (**E**) Quantification of the number of photoreceptor nuclei in a row in transduced and non-transduced organoids at DD210 and (**F**) DD240. (**G**) Quantification of ONL thickness in transduced and non-transduced organoids at DD210 and (**H**) DD240. Each datapoint of the graph represents a single organoid, the value for each organoid is generated from the average value of three independent images at 40× magnification. Error bars represent the standard error of the mean. Unpaired *t*-test, *p* < 0.01 (**); ns: not significant. Number of individual organoids per condition: DD210—untreated *n* = 5; HC-AdV5.F5 *n* = 9; HC-AdV5.F50 *n* = 10; DD240—untreated *n* = 6; HC-AdV5.F5 *n* = 7; HC-AdV5.F50 *n* = 10.

Reactive gliosis is a sign of retinal stress, which can be observed by increased intermediate filament markers such as glial fibrillary acidic protein (GFAP) in Müller glial cells. Immunofluorescence microscopy established that at DD210 and DD240 there was no increase in GFAP expression as a result of transduction with either adenoviral vector type (Figure 6A,B). Additionally, quantification of GFAP signal intensity displayed no significant increase in GFAP within transduced retinal organoids at DD210 and DD240 (Figure 6C,D). Another common sign of retina degeneration is the thinning or swelling of the ONL following or announcing the death of photoreceptors. Therefore, we investigated these aspects by measuring at DD210 and DD240 the number of photoreceptor nuclei in a row in the ONL (Figure 6E,F) and the ONL thickness in µm (Figure 6G,H). The number of photoreceptor nuclei in a row did not statistically differ between non-treated and adenoviral vector-treated specimens. In several independent batches of LUMC0004iCTRL10 iPSC-derived retinal organoids, independently produced using the same differentiation protocol by different team members, we observed an ONL thickness of 28 ± 1 µm at DD225 ± 15 n = 30. At DD210 we observed a trend of increased ONL thickness but without reaching statistical significance between transduced and non-transduced organoids. Notably, the ONL thickness of retinal organoids at DD240 transduced with HC-AdV5.F5 and HC-AdV5.F50 at DD130 was significantly increased by 33.3 % and 44.4%, respectively (*p* < 0.01; 27 ± 2 µm n = 6 non-treated; 36 ± 1 µm n = 7 HC-AdV.F5 and 39 ± 2 µm n = 10 HC-AdV.F50 treated). These data suggest that the two adenoviral vector batches used at a dose of 3.7 × 10^7^ HTU at DD130 caused swelling of the ONL at 110 days post-transduction.

## 3. Discussion

Viral vector delivered gene therapy is an increasingly utilized method for addressing diseases previously considered untreatable. Viral vector delivery of *RPE65* to the retina has met clinically successful endpoints [19], with a number of other promising clinical trials currently underway [20]. AAV has been the vehicle of choice in the first wave of viral vector delivered gene therapies. Although possessing a number of desirable traits, the limited cargo capacity of approximately 4.7 kb precludes the delivery of many large genes. This is of particular importance in the field of retinal gene therapy as a number of coding sequences of clinically relevant genes linked to the required regulatory sequences readily exceeds this limit [4]. In response to this, alternative delivery methods should be explored, with one of these alternative delivery vehicles being high-capacity adenoviral vectors, whose packaging capacity of 36 kb allows for the delivery of all known coding gene sequences found in the human genome [9,21].

To determine transduction endpoints (i.e., spatiotemporal transgene expression profiles) and morphological effects of these vectors with conventional or tropism-modified capsids in the context of the human retina, we utilized human iPSC-derived retinal organoids as a testing platform. Retinal organoids faithfully recapitulate many aspects of the human retina, including the formation of all major retinal cell types arranged in a highly organized stratified architecture [13,14]. HC-AdV vectors have previously been tested ex vivo on cultured human cadaver retinal explants analyzed at 7 days post-transduction, and in vivo in the rat retina [22]. Effective HC-AdV5-CMVp-*eGFP* transduction of human retinal explants was demonstrated, resulting in transgene expression throughout the retina including in the photoreceptors [22]. The 7-day culturing period of the human retinal explants did not allow, however, for intensive analysis of morphological structures. Conversely, in vivo subretinal transduction of HC-AdV on the rat retina revealed structural changes due to an early acute innate inflammatory response to the capsid proteins, including significant upregulation of proinflammatory cytokines and chemokines potentially from Müller glial cells and immune effector cells. Interestingly, inflammatory responses upon HC-AdV exposure were, however, not reported following transduction experiments in the mouse retina [23,24,25,26].

In this study, we investigated for up to 110 days post-transduction in human retinal organoids, the efficiency, tropism and cellular responses of HC-AdV vectors displaying either classical adenovirus type 5 fibers or apical fiber motifs from adenovirus type 50 [15]. The former and latter vector utilize CAR and CD46 as primary receptors, respectively. Of note, CD46-binding vectors bypass the absence of CAR on various human cell types with therapeutic relevant or potential, namely, hematopoietic stem cells [27,28], mesenchymal stromal cells [29] and muscle progenitor cells [15]. In this study, using HC-AdV5.F5 and HC-AdV5.F50 vectors containing the same genome backbone and mCherry reporter, we found that these CAR- and CD46-binding vectors, respectively, are similarly effective gene delivery vehicles in human retinal tissue. The mCherry signals were detected shortly after transduction with live-cell imaging revealing reporter expression as early as 1 day post-transduction. Of note, these initial signals were primarily localized in the RPE, a cell type known for its high phagocytotic activity and ability to readily uptake adenoviral vector and AAV particles [18,30]. The mCherry fluorescence became stronger and more comparable in other regions of the retinal organoid shortly thereafter and, critically, it persisted for up to DD240 (110 days post-transduction) (Figure 2D). Indeed, the transgene expression may have persisted in a sustained fashion even beyond the termination timepoint selected for our experiments.

In addition to general organoid-level transduction analysis, we have also investigated the transduction of retinal cell subtypes, namely, Müller glial and photoreceptor cells, both of which are highly desirable targets for gene therapy. We determined the vector transduction profiles in these cell populations by measuring the colocalization of the mCherry reporter with established cell markers, i.e., CRALBP for Müller glial cells and recoverin for photoreceptors. Our results show a roughly equal capacity of regular and capsid-modified HC-AdV vectors to transduce Müller glial cells when measured at both DD210 and DD240 timepoints. Further, these two adenoviral vector types performed also largely equally well in transducing photoreceptors with approximately 60% of photoreceptors co-expressing the mCherry reporter. Of note, it was observed that several cells within the inner retina also expressed recoverin, which may represent ON-bipolar cells [31] or misplaced photoreceptors. Yet, as we could not define these cells with certainty as being photoreceptors, we limited photoreceptor quantifications to recoverin-positive areas exclusively located in the ONL, where photoreceptor nuclei typically reside.

To address whether high adenoviral vector loads trigger stress responses and degeneration in the retinal organoids, we investigated reactive gliosis which is evidenced by a strong upregulation of the intermediate filament protein GFAP [32]. We compared GFAP levels in untreated organoids versus organoids transduced with HC-AdV5.F5 or HC-AdV5.F50. We found no statistically significant increase in GFAP levels nor did we observe the typical gliosis-associated cell morphology, noting only low GFAP expression in the end feet of Müller glial cells. Additionally, a common sign of retinal degeneration is the loss of photoreceptor cells which can be measured by a thinning of the ONL. Hence, we selected two parameters, the number of ONL nuclei in a row and the length of the ONL thickness in µm. By either metric we did not observe a thinning of the ONL suggesting no increased loss of photoreceptors following transductions with the two HC-AdV constructs. However, at DD240 we did observe a significant increase in ONL thickness following HC-AdV transduction. The swelling of the ONL is not due to changes in the number of photoreceptor nuclei (Figure 6F). Further investigation is needed to reveal if the swelling is due to increased photoreceptor cell volume, increased Müller glial cell volume, extracellular matrix expansion, or expression of mCherry. Regarding the latter, it is of note the existence of experimental evidence indicating that long-term mCherry overexpression is associated with lysosomal accumulation and cytotoxicity in human cells and with abnormal eye development in *Xenopus laevis* [33]. Hence, further research will be necessary to determine whether the ONL thickening at late timepoints post-transduction is the result of HC-AdV exposure per se, long-term mCherry overexpression or a combination of both.

Activated Müller glial cells release inflammatory molecules such as cytokines and chemokines similar to canonical immune effector cells. Inflammatory responses can come from direct Müller glial cell activation or from indirect activation of Müller glial cells via photoreceptor cell damage [34]. Toll-like receptor 9 (TLR9) triggers an early innate immune response to HC-AdV vectors as they sense unmethylated CpG motifs in incoming double-stranded DNA (dsDNA) genomes, which potentially cause cytokine secretion from Müller glial cells in the retinal organoids [35,36]. Besides this TLR9-dependent innate immunity mechanism, there are other early innate immunity restriction factors that could be involved in HC-AdV sensing [37,38]. Other restriction factors include cyclic GMP-AMP synthase (cGAS), which detects cytosolic dsDNA and triggers interferon responses through the interferon regulatory factor-3 (IRF3) stimulator of interferon genes (STING) pathway. These cellular changes in photoreceptors and Müller glial cells may contribute to the increased thickness of the photoreceptor ONL at later stages.

Follow-up work will involve studying innate immune responses directed to DNA and protein components of incoming HC-AdV particles in retinal organoids. A better understanding of these responses might ultimately allow their modulation in the context of clinical HC-AdV delivery of large retinal transgenes. At present, we cannot exclude late-onset effects on the ONL due to toxicity by high levels of mCherry or contaminants in the adenoviral vector preparations. Despite the significant ONL enlargement detected at DD240, transduced organoids developed otherwise normally resulting in a well-structured laminated inner retina, outer plexiform layer, and ONL with visible photoreceptor inner/outer segments arranged in a typical brush border configuration. 

When performing initial HC-AdV dose–response experiments to determine the optimal vector amount, we observed strong and broad transduction of human retinal organoids at 3.7 × 10^7^ HTU when using vectors with either of the ubiquitous promoters tested, i.e., CAG or PGK. In contrast, the use of human cell type-specific promoters such as those of the rhodopsin kinase gene (*GRK1*) and of the retinaldehyde binding protein 1 gene (*RLBP1*) is expected to result in a more defined expression profile in photoreceptors and Müller glial cells, respectively, as previously observed in several studies using AAV vectors [39]. Follow-up work should also exploit the large size of HC-AdV particles to accommodate extensive *cis*-acting regulatory sequences to assure robust and cell type-specific expression of therapeutic transgenes in the retina. Finally, in addition to gene supplementation, the HC-AdV platform can also be in principle directed for delivering large gene-editing tools, such as, prime editing and base editing constructs which, in their full-length formats, cannot be delivered in a single AAV vector [40].

Alternative methods exist for large gene delivery, mostly involving dual AAV vectors encoding transgene halves that, upon co-transduction, lead to mRNA trans-splicing, ribozyme-activated mRNA trans-ligation, Cre-lox DNA sequence-specific and near-unidirectional recombination, or full-length protein assembly via intein-mediated ligation [41,42,43,44]. However, in addition to issues related to the designing complexity and performance of dual AAV vectors, the need to produce at sufficient scale and qualify two independent drug substances increases the challenges and costs of such clinical trials.

In conclusion, here we report that conventional and capsid-modified HC-AdV vectors are capable of achieving robust and persistent transgene expression in human retinal organoids, effectively transducing both Müller glial cells and photoreceptors. Although the observed swelling of the ONL following HC-AdV transduction is a point of concern warranting further research, HC-AdV systems hold promise for the delivery of large genetic payloads to the human retina.

## 4. Materials and Methods

### 4.1. iPSC Culture

The iPSC line LUMC0004iCTRL10 [45] was used for this study. iPSCs were cultured using mTeSR Plus medium (StemCell Technologies, Vancouver, BC, Canada) and Matrigel (Corning, New York, NY, USA)-coated plates in an incubator at 37 °C and 5% CO_2_. Cells were passaged following incubation with Gentle Cell Dissociation Reagent (StemCell Technologies) and mechanical scraping.

### 4.2. Retinal Organoid Differentiation

A modified retinal organoid differentiation protocol was used based on Zhong et al. [13] and Wahlin et al. [14]. To form embryoid bodies (EBs), agarose microwells were generated with 2% agarose solution and MicroTissues 3D Petri Dish micro-mold spheroids (size S, 16 × 16 array, Merck, Darmstadt, Germany) and placed inside 12-well plates. iPSCs were dissociated into single cells using Gentle Cell Dissociation Reagent (StemCell Technologies) and subsequently 1 × 10^6^ cells were transferred to the microwells in a volume of 190 µL of mTeSR Plus medium supplemented with 10 µM blebbistatin. After a period of 10 min to allow the cells to settle in the microwells, 1 mL of mTeSR Plus with 10 µM blebbistatin was added to the area surrounding the microwell in the 12-well plate. The following days the cells were weaned from mTeSR by feeding a mix of mTeSR/neural-induction medium 1 (NIM1) in a ratio of 3:1, 1:1, 0:1 on each subsequent day. As of differentiation day 7 (DD7) EBs were formed and transferred to Matrigel-coated 6-well plates. As of DD21 early organoid structures were manually lifted from the Matrigel and transferred to agarose-coated 6-well plates. Neuroepithelium-like structures were manually sorted from undesired differentiated material and transferred individually to agarose-coated 48-well plates (Figure 1). The medium was changed 3 times per week until the desired collection timepoint. The medium changes schedule was as follows: DD3–10 NIM1, DD10–16 NIM1 containing 100 nM smoothened agonist (SAG), DD16–20 neural-induction medium 2 (NIM2) containing SAG 100 nM, DD20–35 NIM2, DD35–45 retinal lamination medium 1 (RLM1) containing 1 µM retinoic acid (RA), DD44–58 RLM1 containing 1 µM RA and 10 µM gamma secretase inhibitor IX (DAPT), DD58–85 RLM1 with 1 µM RA, DD85–120 retinal lamination medium 2 (RLM2) with 0.5 µM RA, DD120–240 RLM2 without RA. The composition of NIM1, NIM2, RLM1 and RLM2 media was as described previously [13,14,45].

### 4.3. Production and Titration of Adenoviral Vectors

The packaging of reporter HC-AdV genomes into regular and modified AdV capsids was performed in PEC3.30 cells [8] transduced with E1-deleted helper AdV vectors AdV.SRα.lacZ.1.5 and AdV.SRα.lacZ.1.50 to yield CAR-binding and CD46-binding vector particles, respectively [46]. These helpers contain their packaging signals flanked by a direct repeat of loxP site to render them packaging-defective in Cre-expressing producer cells. The subsequent propagation of the resulting reporter HC-AdV particles was performed in helper-transduced PEC.3.30 cells and their purification was performed by sequential buoyant density ultracentrifugation and ultrafiltration steps according to the methodologies detailed elsewhere [40,47]. Of note, Cre-expressing PEC3.30 cells are derived from the adenovirus E1 -complementing AdV packaging cell line PER.C6 [48]. PER.C6 cells were designed to lack overlapping sequence homology between their adenovirus E1 DNA sequences and AdV vector genomes. As such, replication-competent adenovirus (RCA) contaminants characterized by the acquisition of the E1 region through homologous recombination are not generated during vector production [48]. Endpoint titrations of purified HC-AdV stocks were carried out by transducing HeLa cells with a range of vector stock dilutions and, three days post-transduction, the percentages of reporter-positive cells were determined through flow cytometry in a BD LSR II flow cytometer (BD Biosciences, Franklin Lakes, NJ, USA). A minimum of 10,000 viable single cells were acquired per sample with the data being analyzed with the aid of the FlowJo 10.6.1 software (TreeStar, Ashland, OR, USA). The resulting functional vector particle titers are expressed in HeLa cell-transducing units (HTU) per mL. To rule out HC-AdV replication due to the presence of in trans-complementing helper vector particles, the HC-AdV5.F50 vector was applied to highly permissive HeLa cells at saturating multiplicities of infection (MOI). The transduced HeLa cells were subsequently kept for up to 17 days in culture with no evidence for HC-AdV5.F50 replication as traced via the mCherry-directed flow cytometry. Instead, as expected from replication-defective HC-AdV5.F50 episomes, a steep time-dependent decline in the frequency of mCherry-positive cells was observed (Figure S5).

### 4.4. Adenoviral Vector Transduction of Human iPSC-Derived Retinal Organoids

Retinal organoids at DD130 were transferred to an agarose-coated 96-well plate. Individual organoids were subsequently exposed to HC-AdV particles in a volume of 50 µL of RLM2 for a period of 8 h at 5% CO_2_ and 37 °C. The amounts of HC-AdV5.F5 and HC-AdV5.F50 used were 3.7 × 10^5^, 3.7 × 10^6^, and 3.7 × 10^7^ HeLa cell-transducing units (HTU) for comparative analysis. Following the 8-h incubation period the volume of RLM2 was increased to 200 µL. The next day transduced organoids were transferred to a 48-well plate and cultured up to either DD210 or DD240. The HC-AdV particles contained sequences encoding either a mCherry reporter driven by a hybrid CAG promoter [7]; or an EGFP reporter driven by the human *PGK1* gene promoter (HC-AdV5.F50 only) [8].

### 4.5. Immunohistochemical Analysis

Organoids were fixed with 4% paraformaldehyde in PBS for 20 min followed by a brief washing in PBS. Fixed organoids were cryopreserved in 15% sucrose PBS solution for 30 min at room temperature, followed by incubation in a 30% sucrose PBS solution for at least 1 h at room temperature. The organoids were then frozen in Tissue-Tek O.C.T. and stored at −20 °C until further processing. Cryosections were cut at 8 µm thickness using a Leica CM1900 cryostat and transferred to glass slides. Tissue sections were blocked and permeabilized in a solution of 1% BSA, 10% normal goat serum, 0.4% Triton-X in PBS for 30 min. Subsequently, these sections were incubated with primary antibodies, at the appropriate concentrations, overnight at 4 °C in a solution of 1% BSA, 0.4% normal goat serum and 0.04% Triton-X in PBS. Slides were washed twice in PBS before incubation with secondary antibodies in a solution of 1% BSA in PBS for 1 h at room temperature. The slides were subsequently washed twice in PBS before their mounting with Vectashield HardSet Antifade Mounting Medium (Newark, CA, USA) with DAPI. Images were acquired by using a Leica TCS SP8 confocal microscope (Wetzlar, Germany).

The following primary antibodies were used: CRALBP (1:500, Abcam Ab15051, Cambridge, UK); Recoverin (1:1000, Millipore AB5585, Burlington, MA, USA); GFAP (1:200, DAKO Z0334, Golstrup, Denmark). The following secondary antibodies were used: goat anti-mouse or goat anti-rabbit IgGs conjugated to Alexa 488 or Alexa 555 (1:1000, Abcam, Cambridge, UK).

### 4.6. Quantification and Statistical Analysis

Quantification images acquired at 40× magnification, with 3 representative images per organoid being selected. Quantification measurements were made using ImageJ (FIJI, version number 1.53f51). All figure data points represent a single organoid with the value an average of the 3 measurements. The number of organoids (n = 5–10) tested is indicated in the figure legends. Experiments were performed on at least 3 independent rounds of differentiation. Data were normalized to values described in the figure legends. Statistical analysis was performed with Graphpad Prism (Boston, MA, USA, version number 8). The standard error of the mean is derived from the averaged datapoints per individual organoid. Significance is indicated in the graphs as *p* < 0.05 (*), *p* < 0.01 (**), and *p* < 0.001 (***).

## Data Availability

The data that support the findings of this study are available from the corresponding author upon reasonable request.

## Supplementary Materials

The following are available online at: https://www.mdpi.com/article/10.3390/ijms26010055/s1.
