# Supplementary material for: Conventional and Tropism-Modified High-Capacity Adenoviral Vectors Exhibit Similar Transduction Profiles in Human iPSC-Derived Retinal Organoids
Source: Int J Mol Sci. 2024 Dec 24;26(1):55. doi: 10.3390/ijms26010055 (PMC11719574; doi:10.3390/ijms26010055)
Supplement: Supplementary file 1 [file ijms-26-00055-s001.zip › ijms-3354670-supplementary.pdf]

Supplemental figures

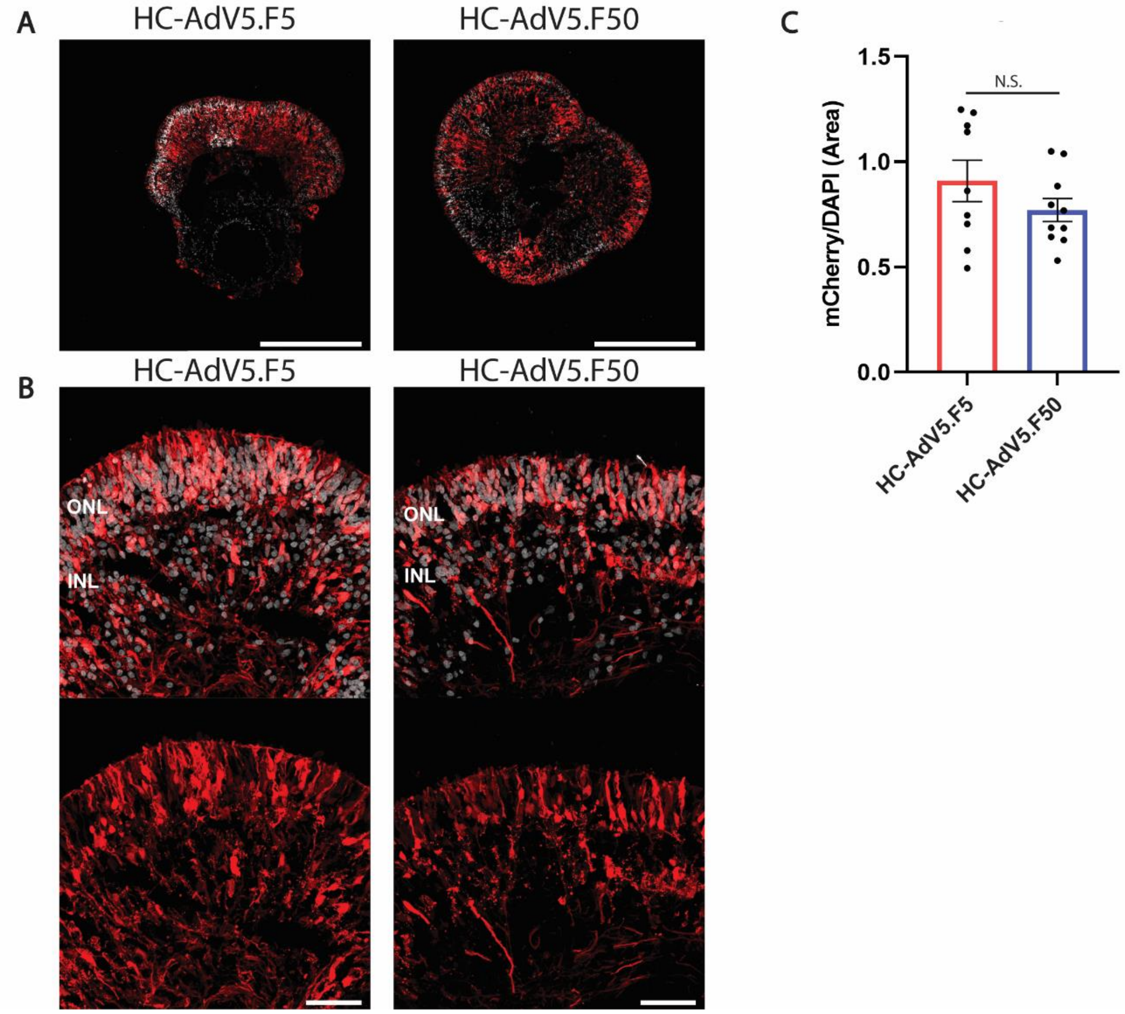

Figure S1. Gene delivery by HC-AdV5.F5 and HC-AdV5.F50 in DD240 human retinal organoids transduced at DD130. A) Representative images of retinal organoids following transduction with HC-AdV5.F5 or HC-AdV5.F50 (10× magnification). Scale bars 500 µm. B) Representative images of retinal organoids transduced with HC-AdV5.F5 or HC-AdV5.F50 (40× magnification). Scale bars 50 µm. C) Quantification of transduction efficiencies achieved by AdV5.F5 and AdV5.F50 calculated as the mCherry-positive area normalized for the DAPI-positive area. Each datapoint of the graph represents a single organoid, with the value for each organoid being generated from the average value of 3 independent images acquired at 40× magnification. Unpaired t-test. Error bars represent standard error of the mean. Number of individual organoids per condition: HC-AdV5.F5 n=9, HC-AdV5.F50 n=10.

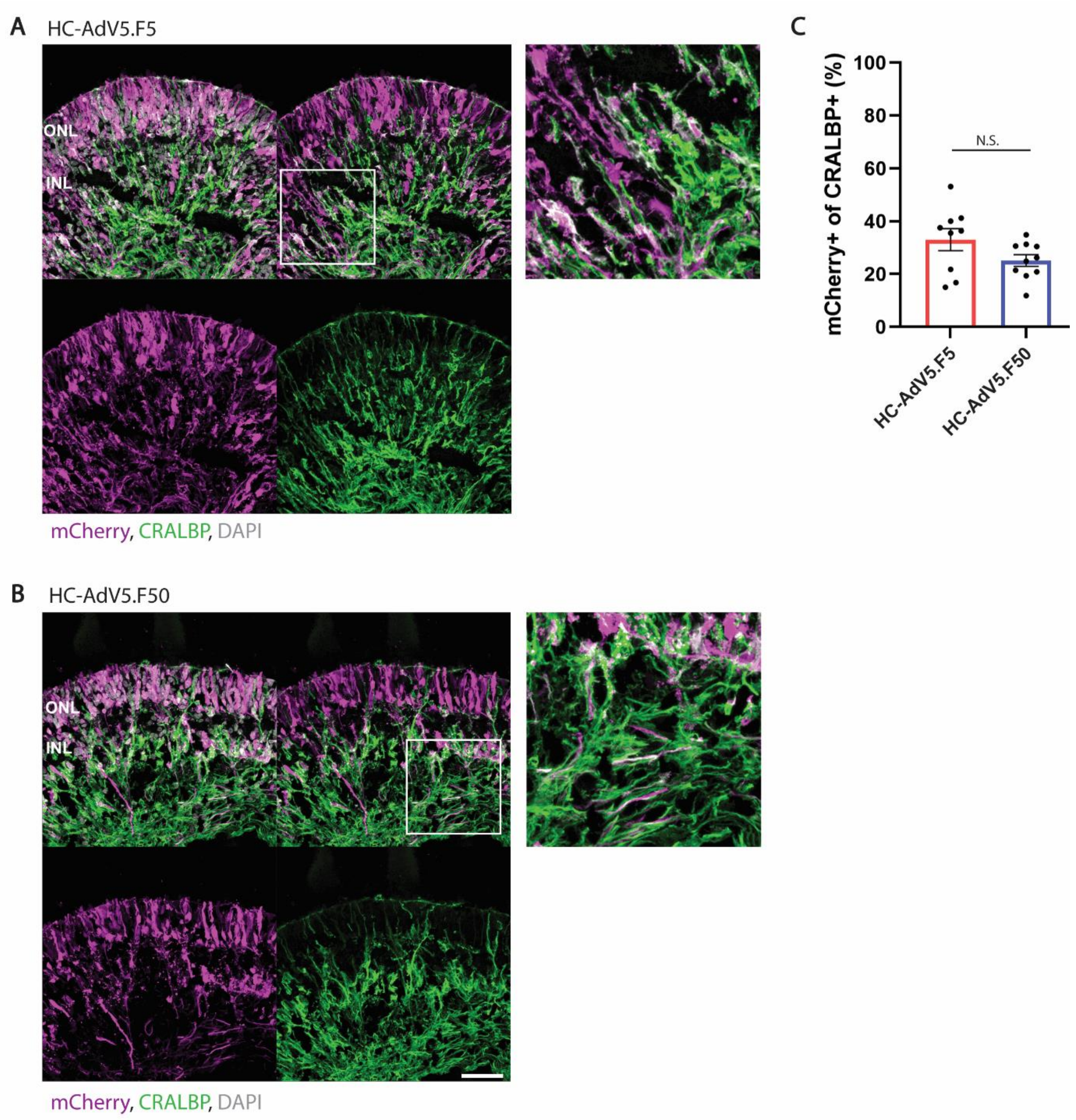

Figure S2. Adenoviral vector transduction of Müller glial cells in DD240 retinal organoids transduced at DD130. A) Representative images of retinal organoids following transduction with HC-AdV5.F5. Colocalization of mCherry and the Müller glial cell marker CRALBP are shown in white. B) Representative images of retinal organoids following transduction with AdV5.F50. Colocalization of mCherry and the Müller glial cell marker CRALBP are shown in white. C) Quantification of transduced Müller glial cells as measured by the percentage of CRALBP-positive areas also expressing mCherry. Scale bars 50 µm. Each datapoint in the graph represents a single organoid, the value for each organoid is generated from the average value of 3 independent images at 40× magnification. Unpaired t-test. Error bars represent standard error of the mean. Number of individual organoids per condition: HC-AdV5.F5 *n*=9, HC-AdV5.F50 *n*=10.

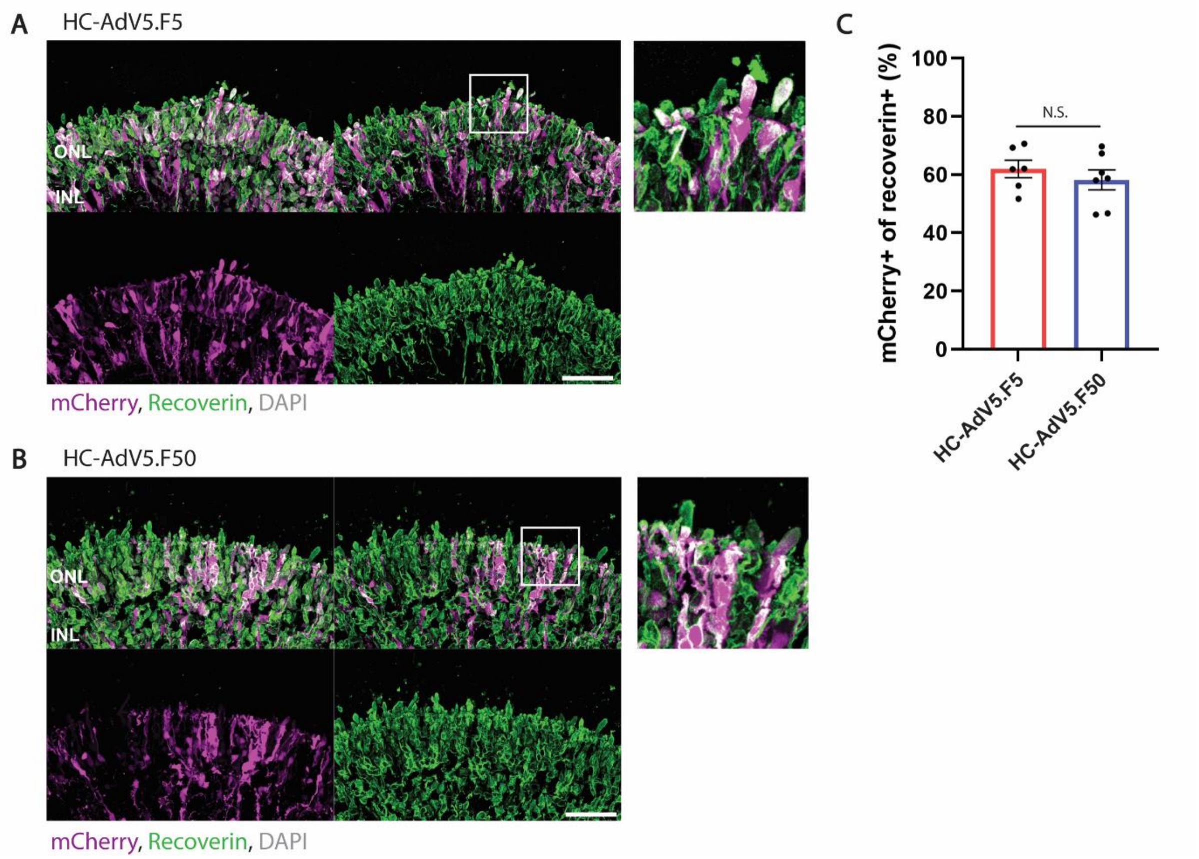

Figure S3. Adenoviral vector transduction of photoreceptors in DD240 retinal organoid transduced at DD130. A) Representative images of retinal organoids following transduction with HC-AdV5.F5. Colocalization of mCherry and the photoreceptor marker recoverin are shown in white. B) Representative images of retinal organoids following transduction with HC-AdV5.F50. Colocalization of mCherry and the photoreceptor marker recoverin are shown in white. C) Quantification of transduced photoreceptors, as measured by the percentage of the recoverin-positive area also expressing mCherry. Scale bars 50 µm. Each datapoint of the graph represents a single organoid, the value for each organoid is generated from the average value of 3 independent images at 40× magnification. Unpaired t-test. Error bars represent standard error of the mean. Number of individual organoids per condition: HC-AdV5.F5 *n*=6, HC-AdV5.F50 *n*=7.

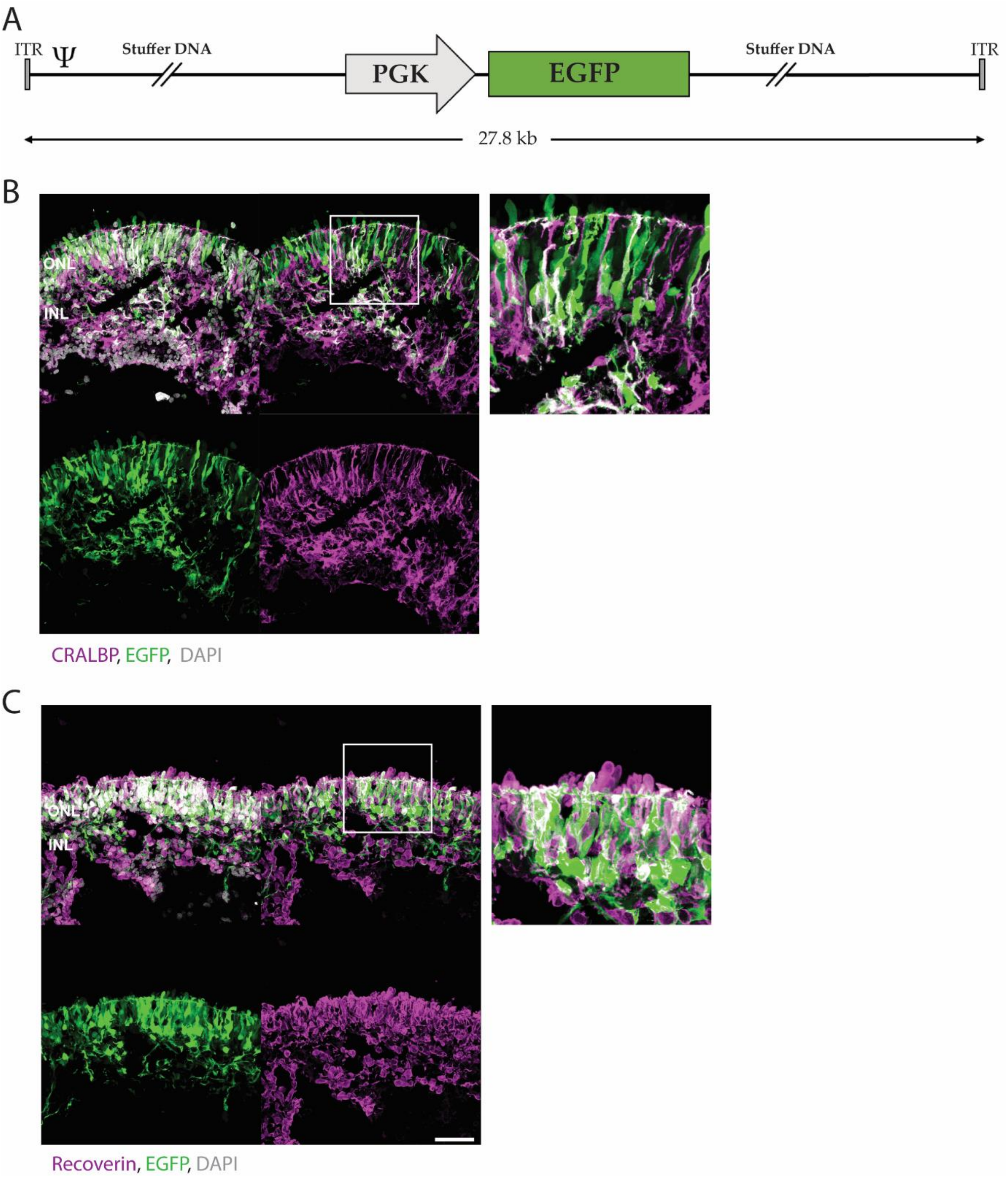

Figure S4. Expression of *EGFP* driven by the PGK promoter in retinal organoids following transduction with HC-AdV5.F50. A) A schematic representation of the transgene delivered by the adenovector, an EGFP fluorophore driven by a ubiquitous PGK promoter. Flanked by stuffer DNA, the entire delivered DNA equals 27.8 kb in length. B) Colocalization of EGFP (green) with the Müller glial cell marker CRALBP (magenta), colocalization in white. C) Colocalization of EGFP (green) with the photoreceptor marker recoverin (magenta) colocalization is shown in white. Organoids were transduced at DD132 and analysed at DD216. Scale bars 50 µm.

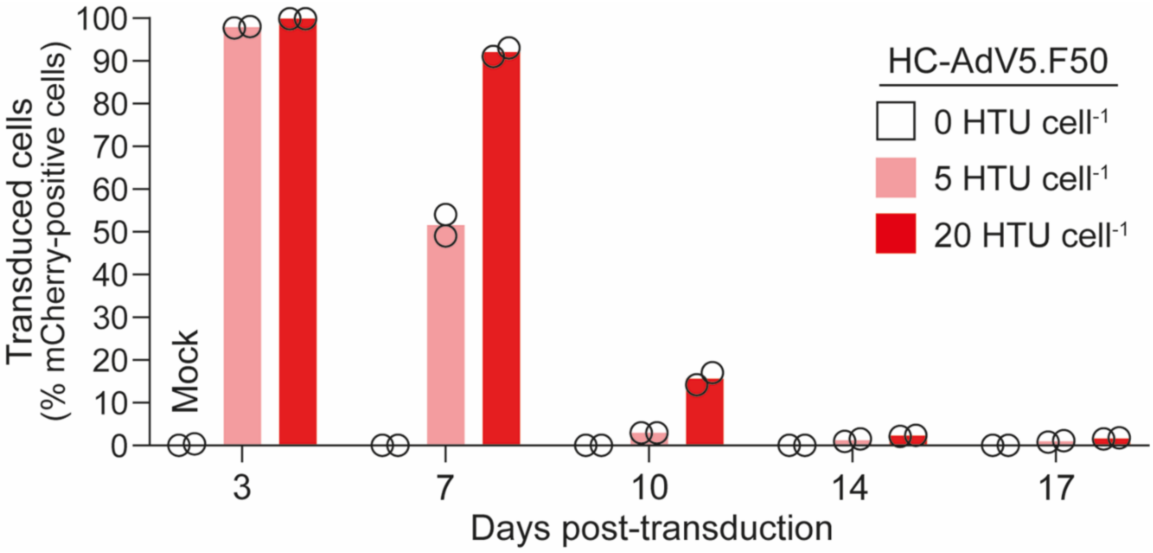

Figure S5. Quantification of high-capacity AdV vector-transduced dividing cells as a function of time. Highly permissive HeLa cells were transduced with HC-AdV5.F50 vector particles at the indicated multiplicities of infection in HeLa cell transducing units per cell (HTU $cell^{-1}$). The percentages of vector-positive cells were determined at the indicated days post-transduction through mCherry-directed flow cytometry (*n*=2 experimental replicates).
